# Associations of Nutritional, Lifestyle, and Metabolic Factors With Non-alcoholic Fatty Liver Disease: An Umbrella Review With More Than 380,000 Participants

**DOI:** 10.3389/fnut.2021.642509

**Published:** 2021-09-17

**Authors:** Yang Xia, Qijun Wu, Huixu Dai, Jiale Lv, Yashu Liu, Hui Sun, Yuting Jiang, Qing Chang, Kaijun Niu, Yuhong Zhao

**Affiliations:** ^1^Department of Clinical Epidemiology, Shengjing Hospital of China Medical University, Shenyang, China; ^2^Clinical Research Center, Shengjing Hospital of China Medical University, Shenyang, China; ^3^Nutritional Epidemiology Institute and School of Public Health, Tianjin Medical University, Tianjin, China

**Keywords:** NAFLD, risk factor, dietary, protective factor, preventive strategy

## Abstract

**Background & Aims:** Nonalcoholic fatty liver disease (NAFLD) is the most common liver injury. We performed this umbrella review of meta-analyses to summarize the evidence on the associations of nutritional, lifestyle, and metabolic factors with NAFLD.

**Methods:** We searched the PubMed, Embase, and Web of Science databases from inception until July 2, 2020, to identify meta-analyses of observational studies which explored the associations of nutritional, lifestyle, and metabolic factors with NAFLD. Evidence levels were assessed using summary effect sizes, 95% prediction intervals, between-study heterogeneity, evidence of small-study effects, and evidence of excess significance bias for each meta-analysis. (No. of PROSPERO, CRD42020200124).

**Results:** Twenty two risk or protective factors from 10 published meta-analyses were included and studied. Three risk factors (sugar-sweetened beverage consumption, serum fetuin-A, and waist circumference) with highly suggestive levels of evidence and three risk factors (soft drink consumption, former smoking, and body mass index) with suggestive levels of evidence were identified. Only two protective factors (physical activity and serum vitamin D level [among adults in Western countries]) with suggestive levels of evidence were identified. Furthermore, other six risk factors and two protective factors with weak levels of evidence were identified.

**Conclusions:** We found varying levels of evidence of associations of nutritional, lifestyle, and metabolic factors and NAFLD. The results suggest that nutritional and lifestyle management should be considered as a major primary preventive strategy for NAFLD. Moreover, considering the low quality of included meta-analyses and limited area of research topics, future high-quality original studies and meta-analyses should be performed to study these associations.

## Introduction

Non-alcoholic fatty liver disease (NAFLD) has variable disease courses, including simple steatosis, non-alcoholic steatohepatitis, and cirrhosis (1). NAFLD not only leads to advanced liver disease such as hepatic cancer (2), but also is associated with metabolic syndrome (3), type 2 diabetes (4), and cardiovascular disease (5). Of note, NAFLD is the most common form of liver injury with a global prevalence of 25% (6). There has been a rapid increase in the prevalence of NAFLD in recent decades. For example, the prevalence of NAFLD in Asia increased from 25.28% (95% confidence interval [CI], 22.42–28.37) between 1999 and 2005 to 33.90% (95% CI, 31.74–36.12) between 2012 and 2017 (7). In parallel with the worsening epidemic of NAFLD, the economic burden of this disease was high. A previous study estimated that the annual direct medical costs attributable to NAFLD were $103 billion ($1,613 per patient) in the United States and €35 billion (€354 to €1,163 per patient) in the “Big Four of Europe” (8). Considering the rising epidemic, the associated complications, and the high economic burden of NAFLD, targeted prevention strategies are needed.

A growing body of evidence suggested that both genetic and environmental factors (e.g., diet and physical activity) contribute to the development of NAFLD (9–11). Moreover, metabolic factors (e.g. obesity) also play important roles in the development of NAFLD (12). Compared with genetic factors, environmental and metabolic factors are more modifiable by preventive interventions. Therefore, it is necessary to understand the role of nutritional, lifestyle, and metabolic factors for NAFLD and raise awareness among the general public. Numerous meta-analyses and systematic reviews of epidemiological studies have found that nutritional, lifestyle, and metabolic factors are associated with NAFLD [e.g. dietary intake (13), physical activity (13), and smoking (14)]. However, before interventional strategies for NAFLD are implemented, it is important to assess the validity of the associations between such strategies and NAFLD. Moreover, previous efforts to systematically appraise the evidence on NAFLD have been focused on single effect factor (15). An umbrella review provides the opportunity to conclude on the overall evidence from previous meta-analyses with strict criteria and procedures (16). Moreover, an umbrella review can generate hierarchical pieces of evidence across diverse nutritional, lifestyle, and metabolic factors associated with NAFLD and provide complex interpretations of these associations.

To the best of our knowledge, no umbrella review has systematically concluded on the overall associations between diverse nutritional, lifestyle, and metabolic factors (risk or protective) and NAFLD. To fill this gap in the literature, we performed this umbrella review to summarize the results of existing meta-analyses that have explored the associations between nutritional, lifestyle, and metabolic factors and NAFLD in both adults and children. Further, we assessed the robustness of the evidence to inform future preventive strategies.

## Methods

We followed a standardized methodology and reported the findings of this umbrella review according to the recommendations of the Meta-analyses of Observational Studies in Epidemiology recommendations (17). The protocol of the present study has been registered in PROSPERO (CRD42020200124).

### Search Strategy

To identify systematic reviews or meta-analyses of observational studies that examined the associations of a number of nutritional, lifestyle, and metabolic factors with NAFLD, we systematically and comprehensively searched the PubMed, Embase, and Web of Science databases from their inception to July 2, 2020, without restrictions. The search keywords of each database are provided in Supplementary Table 1. Furthermore, we manually searched the references of relevant articles and attempted to identify and include eligible studies.

### Inclusion and Exclusion Criteria

Publications were initially screened based on the title and abstract. The full texts of potentially eligible publications were scrutinized independently by two investigators (H-XD and Y-SL). If these two investigators disagreed, a third investigator (Q-JW) were applied to make the final decision. Studies were included if they met the following criteria: (1) meta-analyses of any one of the observational study designs (e.g., as a cohort study, case-control study, cross-sectional study, or ecological studies); (2) the exposure of interest as nutritional, lifestyle, and metabolic factors; (3) reporting on the NAFLD as the outcome; (4) reporting of the usable risk estimates (e.g., risk ratios [RRs], odds ratios [ORs], hazard ratios [HRs], and incidence rate ratios or necessary data for calculation) between nutritional, lifestyle, and metabolic factors and NAFLD; (5) published in the English; (6) including both adults and children. Articles that described separate meta-analyses of more than one nutritional, lifestyle, and metabolic factors were included separately. Furthermore, for more than one meta-analysis on the same association, we included the one which assessed the largest number of primary studies. For meta-analyses on the same association that included the same number of primary studies, we included the one with the largest amount of prospective data.

We excluded meta-analyses that investigated the association between genetic markers and the risk of NAFLD. Trials were unavailable for our research question. We excluded systematic reviews that did not feature quantitative analyses, meta-analyses based on individual data without systematic review, or articles that included animal trials or laboratory studies. We also excluded systematic reviews or meta-analyses that lacked study-specific data (risk estimates, the number of cases and controls, or total study population size). In addition, studies that examined NAFLD as a risk factor for other medical conditions or diseases were also excluded.

### Data Extraction

For each eligible meta-analysis, we extracted the name of the first author, journal name, publication year, study design, number of studies included, study population, nutritional, lifestyle, and metabolic factors, outcome(s) of interest investigated, and type of effect metric. Moreover, we extracted information on each primary study in the included meta-analyses, including the exposure, first author, publication year, the number of cases and controls (case-control studies), participants, the length of follow-up (cohort studies), risk estimates, and 95% CIs. We also extracted information relating to dose-response relationships from all meta-analyses. We included studies reporting different measures of effect size (ES) (RRs, ORs, HRs, and incidence rate ratios). Missing measures were calculated if sufficient data were available. Two independent investigators (J-LL and HS) extracted data from eligible publications. Discrepancies between the data extracted by the two investigators were resolved by a third investigator (Q-JW).

### Risk of Bias Assessment

The investigators (Y-TJ and YX) independently assessed the methodological quality of qualified systematic reviews and meta-analyses using AMSTAR 2 (A Measurement Tool to Assess systematic Reviews) (18). Discrepancies were resolved through discussion with a third investigator (Q-JW). The instrument assesses the overall rating of 16 items related to weaknesses in critical domains. In addition, AMSTAR 2 rates the methodological quality of reviews as high, moderate, low, or critically low, instead of yielding an overall score (18).

### Statistical Analysis

For each meta-analysis, we calculated the summary ES, along with 95% CIs and *P* values, using both fixed- and random-effects models (19). Between-study heterogeneity was assessed using *I*^2^ statistic (20). We also assessed the uncertainty surrounding heterogeneity estimates by calculating 95% CIs and *P* values. *I*^2^ values of 50% or more were considered to represent high levels of heterogeneity, whereas values exceeding 75% were considered to represent very high levels of heterogeneity. The 95% prediction intervals (PIs) for the random-effects estimates were calculated to account for between-study heterogeneity and to represent the possible range in which the risk estimates of new studies might lie (21). Egger's regression asymmetry test was also performed to determine small-study effects. A *P* value of < 0.10 arising from Egger's test was considered to represent evidence of small-study effects (22). We evaluated excess significance bias by investigating whether the observed (O) number of nominally significant study findings were significantly different from the expected (E) number of statistically significant study findings. To do this, we performed a chi-squared test to compare the difference between O and E values (23). The ES of the largest study in each meta-analysis was used to determine the power estimates for each component of a particular study using a non-central *t* distribution (23). Excess statistical significance for a single meta-analysis was determined by a *P* value < 0.10 and if O > E (23). Statistical analyses were performed using Stata version 12.0 (StataCorp LLC, College Station, TX, USA) and all *P* values were two-tailed. Using the methodology described above, and in accordance with previous published umbrella reviews (24–27), we categorized the strength of the evidence of nutritional, lifestyle, and metabolic factors for NAFLD into convincing, highly suggestive, suggestive, weak evidence, or non-significant associations as follows:

1. The evidence was defined as convincing when the *P* value of the random-effects model was smaller than 10^−6^, the meta-analysis included more than 1,000 cases or more than 20,000 participants for continuous outcomes, if the largest component study in the meta-analysis reported a significant result (*P* < 0.05), if the 95% PIs excluded the null hypothesis, if the I^2^ statistic for heterogeneity was <50%, if there was no evidence of small study effects (*P* > 0.10), and if excess significance bias (*P* > 0.10) was indicated.
2. The evidence was defined as highly suggestive if the *P* value for the random-effects model was <10^−6^, if the meta-analysis included more than 1,000 cases or more than 20,000 participants for continuous outcomes, and if the largest component study reported a significant result.
3. The evidence was defined as suggestive if the *P* value for random-effects was <10^−3^, or if there were more than 1,000 cases or more than 20,000 participants for continuous outcomes.
4. The evidence was defined as weak if the *P* value for significant associations was <0.05.
5. We used the ‘non-significant associations' classification if all association tests yielded a *P* value > 0.05.

## Results

### Search Results

**Figure 1:**
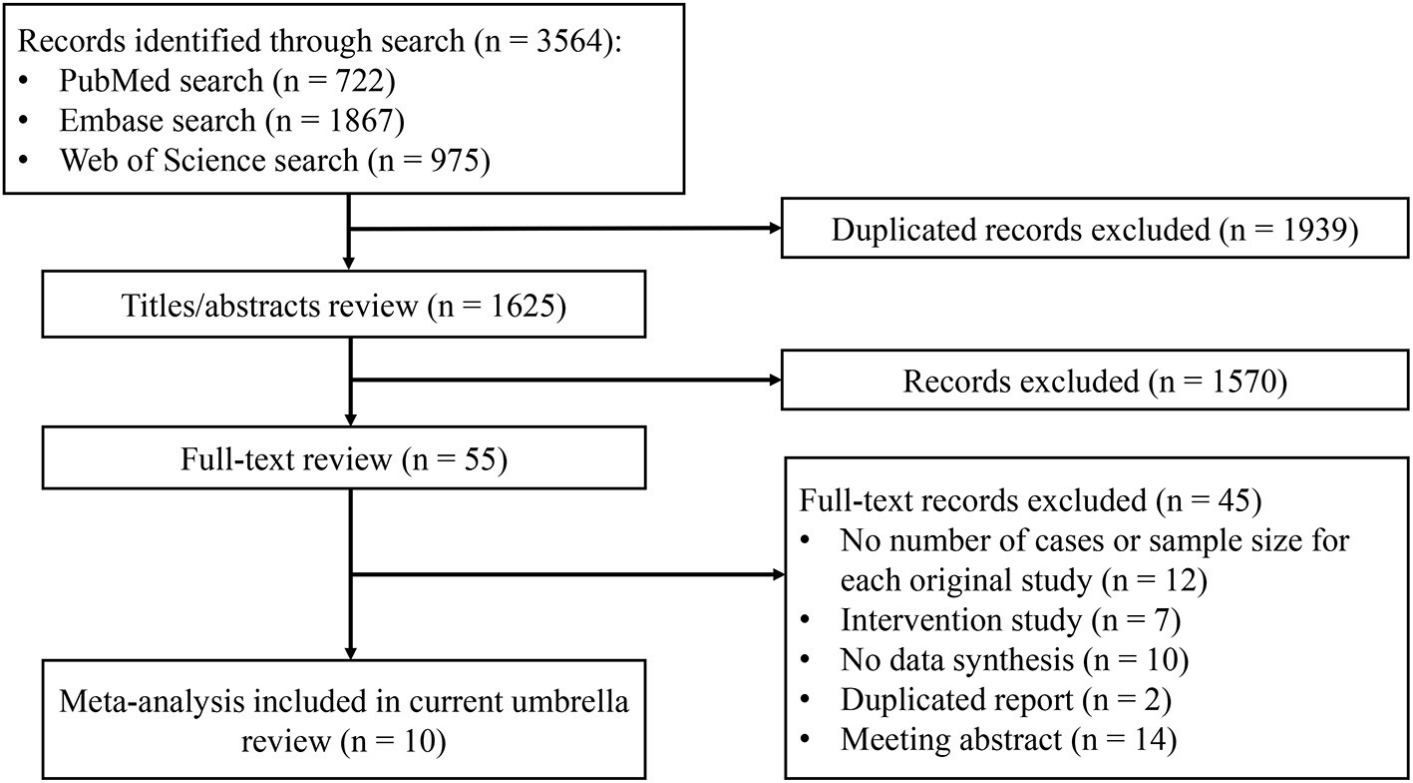
Flowchart of selection of meta-analyses for inclusion in umbrella review.

**Figure 2:**
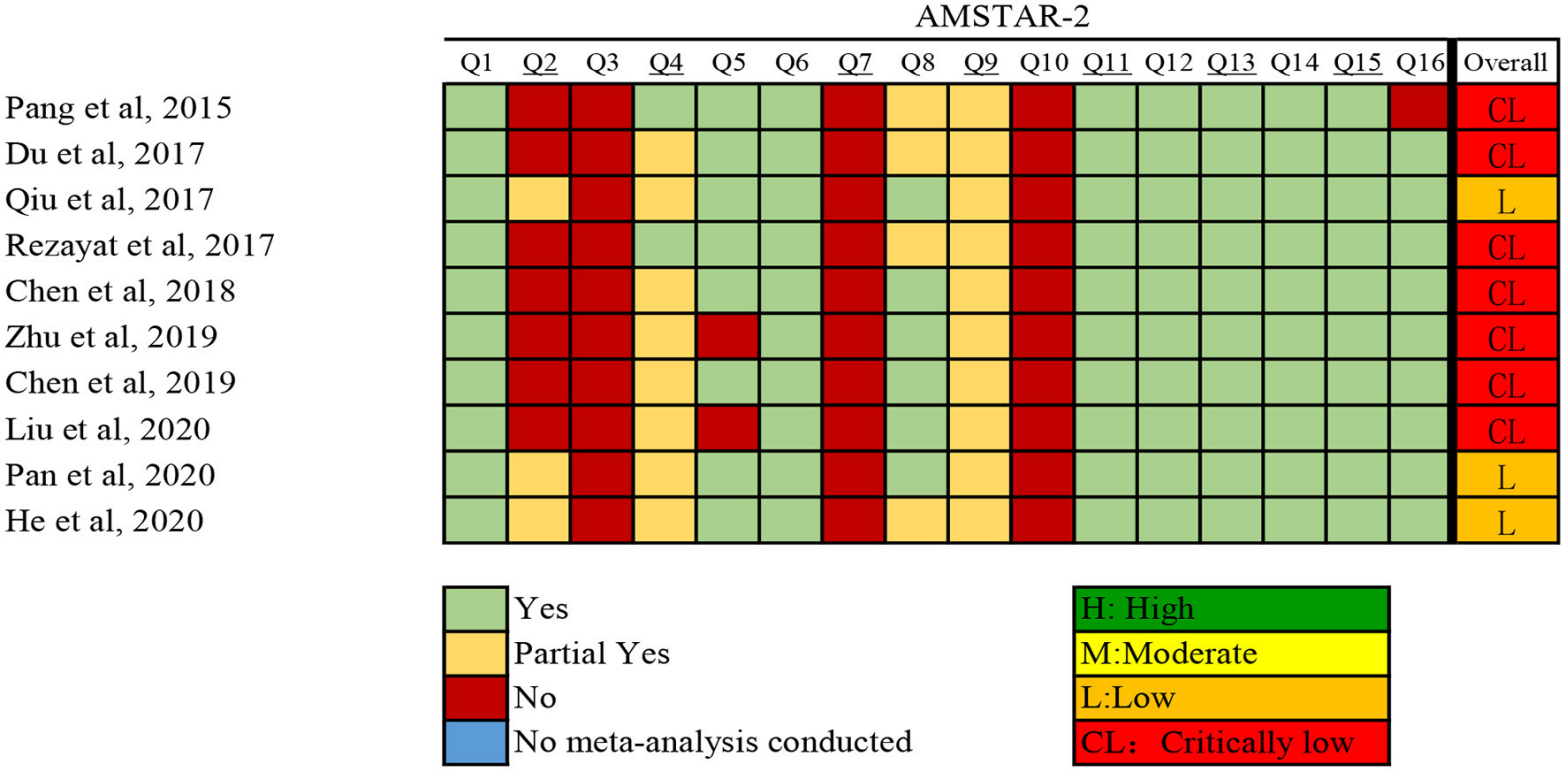
The AMSTAR 2 rating of the included articles.

The results of the systematic search and selection of eligible studies are presented in Figure 1. Overall, 3,564 articles were searched and 10 meta-analyses published from 2014 to 2020 (13, 14, 28–35) were eligible. The median number of studies per meta-analysis included was 13 (range, 7–30). Detailed descriptions of the 10 articles included are presented in Supplementary Table 2. The articles excluded during the process of full text review are shown in Supplementary Table 3. Overall, 22 risk or protective factors (including dietary intake, lifestyle, biomarkers, and metabolic factors) of NAFLD were reported (Table 1). The median number of total included participants and cases for each factor was 27,396 (rang, 1,267-142,781) and 6,126 (rang, 519–32,657), respectively. A total of 382,199 (85,742 cases) participants were included. The AMSTAR 2 ratings of the included articles are presented in Figure 2. As shown, all articles included were rated as low (*n* = 3, 25%) or critically low (*n* = 7, 70%).

**Table 1:** Characteristics and quantitative synthesis of the eligible meta-analyses of multiple risk factors for NAFLD.

**Factor**	**No. of studies**	**No. of cases/participants**	**Level of comparison**	**Summary effect size (95% CI)**			**Random *P* value^†^**	**Fixed *P* value^‡^**
				**Random effects**	**Fixed effects**	Largest study*		
**Dietary intake**								
Coffee intake	7	3,705/42,052	<1 vs. 1–2 cups/day	0.97 (0.85–1.12)	1.00 (0.95–1.06)	1.00 (0.92–1.09)	0.700	0.98
	7	3,400/39,007	<1 vs. >2 cups/day	0.88 (0.73–1.06)	0.96 (0.90–1.01)	1.01 (0.92–1.09)	0.18	0.12
	7	4,825/54,441	Dose-response	0.96 (0.91–1.02)	0.95 (0.92–0.98)	0.93 (0.89–0.96)	0.21	0.001
Sugar-sweetened beverages	12	9,434/35,705	Exposed vs. unexposed	1.48 (1.29–1.69)	1.39 (1.29–1.49)	1.26 (1.14–1.39)	1.570 × 10^−8^	3.714 × 10^−18^
Red meat	7	5,141/12,946	High vs. low	1.22 (1.06–1.41)	1.12 (1.04–1.21)	1.04 (0.95–1.14)	0.005	0.002
Soft drinks	6	9,887/37,320	High vs. low	1.32 (1.17–1.48)	1.29 (1.19–1.41)	1.26 (1.14–1.40)	6.056 × 10^−6^	1.084 × 10^−9^
Nuts	5	5,505/26,386	High vs. low	0.87 (0.78–0.98)	0.94 (0.91–0.97)	0.95 (0.91–0.98)	0.03	0.001
Refined grains	6	3,716/11,414	High vs. low	1.01 (0.95–1.08)	1.01 (0.99–1.02)	1.01 (0.99–1.03)	0.75	0.61
Fish	5	2,780/5,369	High vs. low	0.91 (0.65–1.28)	0.98 (0.89–1.08)	1.01 (0.91–1.12)	0.58	0.73
Fruits	8	14,029/71,796	High vs. low	0.96 (0.84–1.09)	1.01 (0.96–1.06)	1.02 (0.96–1.08)	0.55	0.67
Vegetables	8	7,597/44,293	High vs. low	0.97 (0.89–1.05)	1.00 (0.98–1.03)	1.01 (0.98–1.04)	0.47	0.76
Dairy	3	6,789/28,686	High vs. low	0.95 (0.82–1.10)	0.95 (0.88–1.04)	1.02 (0.92–1.14)	0.52	0.26
Legumes	3	2,614/4,881	High vs. low	0.94 (0.88–1.01)	0.94 (0.88–1.01)	0.95 (0.88–1.02)	0.12	0.12
**Lifestyle**								
Physical activity	5	32,657/142,781	Highest vs. lowest	0.79 (0.71–0.89)	0.82 (0.78–0.87)	0.86 (0.80–0.92)	7.160 × 10^−5^	9.183 × 10^−13^
Smoking	12	5,972/20,149	Exposed vs. unexposed	1.09 (0.97–1.23)	1.10 (1.02–1.19)	1.07 (0.93–1.24)	0.16	0.01
Current smoking	4	1,350/4,754	Exposed vs. unexposed	1.04 (0.83–1.32)	1.03 (0.90–1.19)	1.05 (0.88–1.25)	0.72	0.64
Former smoking	4	1,579/5,075	Exposed vs. unexposed	1.32 (1.16–1.50)	1.32 (1.16–1.50)	1.32 (1.11–1.55)	2.538 × 10^−5^	2.538 × 10^−5^
**Biomarker**								
Serum ferritin	3	519/1,267	Dose-response	4.98 (1.95–12.73)	6.30 (5.05–7.85)	6.60 (5.12–8.35)	0.001	6.817 × 10^−60^
Serum fetuin-A	19	1,594/3,658	Dose-response	3.22 (2.03–5.11)	2.66 (2.33–3.05)	1.52 (1.18–1.99)	6.730 × 10^−7^	2.416 × 10^−45^
	8 (only NASH)	515/908	Dose-response	10.63 (3.85–29.33)	5.95 (4.50–7.86)	1.66 (0.88–3.14)	5.078 × 10^−6^	4.481 × 10^−36^
Serum fetuin-B	4	1,691/2,848	Dose-response	1.38 (1.05–1.82)	1.36 (1.19–1.56)	1.29 (1.06–1.57)	0.02	9.225 × 10^−6^
Serum vitamin D	8 (adults in western countries)	1,524/4,389	Standard vs. deficient	0.60 (0.46–0.77)	0.79 (0.78–0.81)	0.52 (0.51–0.54)	1.052 × 10^−4^	6.530 × 10^−185^
	7 (adults in eastern countries)	6,279/15,707	Standard vs. deficient	0.70 (0.55–0.88)	0.88 (0.84–0.93)	0.94 (0.88–0.99)	0.003	9.909 × 10^−7^
	8 (children and adolescents)	524/2,052	Dose-response	0.34 (0.17–0.69)	0.39 (0.32–0.48)	0.47 (0.34–0.64)	0.003	1.881 × 10^−19^
**Metabolic factors**								
Waist circumference	9	9,909/34,643	Dose-response	1.08 (1.03–1.13)	1.07 (1.05–1.09)	1.07 (1.03–1.11)	0.002	8.056 × 10^−11^
	7	1,224/4,753	High vs. low	2.55 (1.80–3.62)	2.35 (1.83–3.00)	1.76 (1.12–2.76)	1.466 × 10^−7^	1.459 × 10^−11^
Waist-to-hip ratio	3	387/1,063	High vs. low	4.06 (1.52–10.80)	3.91 (2.25–6.77)	3.29 (1.52–7.11)	0.005	1.239 × 10^−6^
Body mass index	9	9,909/34,643	Dose-response	1.31 (1.17–1.46)	1.21 (1.17–1.25)	1.11 (1.05–1.17)	3.065 × 10^−6^	2.463 × 10^−26^
	5	705/3,527	High vs. low	2.86 (1.61–5.08)	2.19 (1.57–3.03)	1.49 (0.97–2.28)	3.541 × 10^−4^	1.973 × 10^−6^

*CI, confidence interval; NAFLD, non-alcoholic fatty liver disease; NASH, non-alcoholic steatohepatitis*.

† *P value of summary random effects estimate*.

‡ *P value of summary fixed effects estimate. All statistical tests were two-sided*.

### Dietary Intake and NAFLD

**Figure 3:**
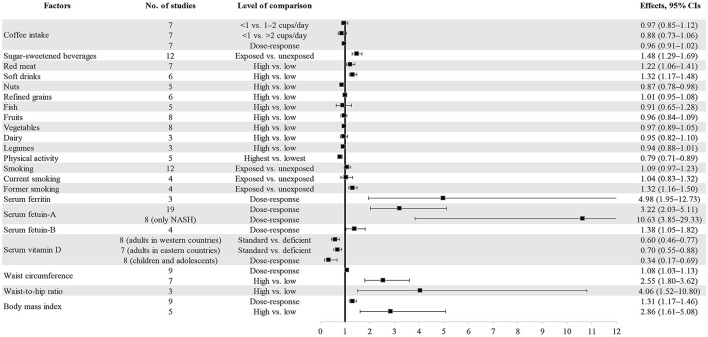
Forest plot of summary effect sizes of multiple risk factors for NAFLD with random-effects model.

Four out of 11 dietary factors were found to be associated with NAFLD (Tables 1, 2). Intakes of sugar-sweetened beverages (evidence class, highly suggestive; random effect ES [95% CI], 1.48 [1.29–1.69]), soft drinks (evidence class, suggestive; random effect ES [95% CI], 1.32 [1.17–1.48]), and red meat (evidence class, weak; random effect ES [95% CI], 1.22 [1.06–1.41]) were determined as risk factors, whereas intake of nuts (evidence class, weak; random effect ES [95% CI], 0.87 [0.78–0.98]) was determined as a protective factor for NAFLD. There were no associations between other dietary factors and NAFLD (Figure 3).

**Table 2:** Levels of evidence for the association of risk factors for NAFLD.

**Factor**	**Features used for classification of the level of evidence**								**Evidence classification**
	**Significance threshold reached**	**I (95% CI)**	**95% prediction interval**	**Egger's *P* value**	**Excess significance^§^**		**Largest study significant**	**Small-study effect/excess significant bias**	
					**O/E^#^**	***P value^¶^***			
**Food**									
Coffee intake	>0.05	74.3% (45–88%)	0.65–1.47	0.67	3/2.56	0.73	No	No / No	No association
	>0.05	86.8% (75–93%)	0.47–1.64	0.40	2/2.09	NP	No	No / No	No association
	>0.05	60.5% (9–83%)	0.81–1.14	0.65	3/2.33	0.59	Yes	No / No	No association^**^
Sugar-sweetened beverages	<10	42.2% (0–71%)	1.06–2.06	0.43	7/3.51	0.03	Yes	No / Yes	Highly suggestive
Red meat	<0.05 but >0.001	48.7% (0–78%)	0.85–1.76	0.003	4/2.20	0.14	No	Yes / No	Weak
Soft drinks	<0.001 but >10	25.5% (0–69%)	1.01–1.72	0.52	4/1.16	0.003	Yes	No / Yes	Suggestive
Nuts	<0.05	40.9% (0–78%)	0.63–1.20	0.007	2/1.12	0.34	Yes	Yes / No	Weak
Refined grains	>0.05	71.4% (34–88%)	0.85–1.21	0.98	2/2.38	NP	NO	No / No	No association
Fish	>0.05	69.7% (22–88%)	0.30–2.71	0.63	1/1.74	NP	No	No / No	No association
Fruits	>0.05	68.4% (34–85%)	0.68–1.36	0.26	2/2.89	NP	No	No / No	No association
Vegetables	>0.05	30.9% (0–69%)	0.81–1.16	0.05	2/1.53	0.67	No	Yes / No	No association
Dairy	>0.05	55.7% (0–87%)	0.21–4.28	0.89	1/0.87	0.87	No	No / No	No association
Legumes	>0.05	0 (0–90%)	0.59–1.51	0.28	0/0.31	NP	No	No / No	No association
**Lifestyle**									
Physical activity	<0.001 but >10	59.1% (0–83%)	0.57–1.09	0.49	4/2.09	0.08	Yes	No / Yes	Suggestive
Smoking	>0.05	41.0% (0–70%)	0.81–1.47	0.64	1/2.87	NP	No	No / No	No association
Current smoking	>0.05	49.6% (0–83%)	0.44–2.48	0.76	1/0.91	0.91	No	No / No	No association
Former smoking	<0.001 but >10	0 (0–85%)	0.99–1.74	0.78	2/0.33	0.003	Yes	No / Yes	Suggestive
**Biomarkers**									
Serum ferritin	<0.05	88.2% (67–96%)	0.00–503018.66	0.74	2/1.42	0.50	Yes	No / No	Weak^**^
Serum fetuin-A	<10	90.0% (86–93%)	0.42–24.91	0.26	14/9.38	0.03	Yes	No / Yes	Highly suggestive^**^
	<0.001 but >10	91.8% (87–95%)	0.28–409.75	0.13	6/5.64	0.78	No	No / No	Weak^**^
Serum fetuin-B	<0.05	61.9% (0–86%)	0.59–3.21	0.96	3/1.39	0.09	Yes	No / Yes	Weak^**^
Serum vitamin D	<0.001 but >10	99.5% (99–100%)	0.25–1.43	0.79	6/4.34	0.24	Yes	No / No	Suggestive
	<0.05 but >0.001	87% (76–93%)	0.33–1.49	0.08	4/3.33	0.61	Yes	Yes / No	Weak
	<0.05 but >0.001	89.7% (82–94%)	0.03–4.06	0.61	6/4.04	0.16	Yes	No / No	Weak
**Metabolic factors**									
Waist circumference	<0.05 but >0.001	73.2% (48–86%)	0.93–1.25	0.21	7/2.75	0.002	Yes	No / Yes	Weak^**^
	<10	41.7% (0–75%)	1.05–6.18	0.09	6/2.09	0.001	Yes	Yes / Yes	Highly suggestive
Waist-to-hip ratio	<0.05 but >0.001	65.9% (0–90%)	0.00–227578.72	0.86	2/0.85	0.14	Yes	No / No	Weak
Body mass index	<0.001 but >10	83.9% (71–91%)	0.92–1.85	0.08	7/5.04	0.19	Yes	Yes / No	Suggestive^**^
	<0.001 but >10	57.5% (0–84%)	0.47–17.36	0.01	3/1.93	0.32	No	Yes / No	Weak

*CI, confidence interval; NAFLD, non-alcoholic fatty liver disease*.

§ *Expected number of statistically significant studies using the point estimate of the largest study (smallest standard error) as the plausible effect size*.

# *Observed/expected number of statistically significant studies*.

¶ *P value of the excess statistical significance test*.

^*^P value under the random-effects model

** *Exposure as a continuous variable*.

### Lifestyle and NAFLD

As shown in Tables 1, 2, two out of four lifestyle factors were associated with NAFLD. Physical activity was inversely associated with NAFLD (evidence class, suggestive; random effect ES [95% CI], 0.79 [0.71–0.89]). Former smoking was positively associated with NAFLD (evidence class, suggestive; random effect ES [95% CI], 1.32 [1.16–1.50]). We found no association between smoking/current smoking and NAFLD (Figure 3).

### Biomarkers of NAFLD

We explored the associations between the levels of four blood biomarkers and NAFLD (Tables 1, 2). Serum ferritin level was found to be a risk factor for NAFLD (evidence class, weak; random effect ES [95% CI], 4.98 [1.95–12.73]). Serum fetuin level was determined to be a risk factor for NAFLD. The evidence classes of the ESs for serum fetuin-A (random effect ES [95% CI], 3.22 [2.03–5.11]) and serum fetuin-B (random effect ES [95% CI], 1.38 [1.05–1.82]) were determined as highly suggestive and weak, respectively. The vitamin D level was determined as a protective factor for NAFLD. The evidence classes of the ESs for vitamin D among adults in western countries (random effect ES [95% CI], 0.60 [0.46–0.77]), adults in eastern countries (random effect ES [95% CI], 0.70 [0.55–0.88]), and children/adolescents (random effect ES [95% CI], 0.34 [0.17–0.69]) were suggestive, weak, and weak, respectively (Figure 3).

### Metabolic Factors

Waist circumference, body mass index (BMI), and waist-to-hip ratio were all determined as metabolic risk factors for NAFLD (Tables 1, 2). For waist circumference, the ESs of high vs. low (random effect ES [95% CI], 2.55 [1.80–3.62]) and dose-response (random effect ES [95% CI], 1.08 [1.03–1.13]) associations were classified as highly suggestive and weak, respectively. For BMI, the ESs of high vs. low (random effect ES [95% CI], 2.86 [1.61–5.08]) and dose-response (random effect ES [95% CI], 1.31 [1.17–1.46]) associations were classified as weak and suggestive, respectively. For the waist-to-hip ratio, the ES of a high vs. low associations (random effect ES [95% CI], 4.06 [1.52–10.80]) was classified as weak (Figure 3).

## Discussion

To the best of our knowledge, our study is the first umbrella review that provides a comprehensive overview and critical assessment of risk and protective factors for NAFLD. A total of 22 risk or protective factors (including dietary intake, lifestyle, biomarkers, and metabolic factors) from 10 published meta-analyses were included and studied. Regarding risk factors, three factors (consumption of sugar-sweetened beverages, serum fetuin-A, and waist circumference) with highly suggestive levels of evidence and three factors (consumption of soft drinks, former smoking, and BMI) with suggestive levels of evidence were identified. Only two protective factors (physical activity and serum vitamin D level [among adults in western countries]) with suggestive levels of evidence were identified. Furthermore, six risk factors and two protective factors with weak levels of evidence were also identified. This umbrella review summarized the results based on existing meta-analyses. However, many novel and latest factors are not included in this study because of few meta-analyses of these topic. For example, pervious study found that seaweed consumption (36). intake of insoluble dietary fiber (37), yogurt consumption (38), mushroom intake (39), and consuming honey 2-6 times/week (40) were inversely associated with NAFLD. For lifestyle, faster speed of eating, overall computer/mobile devices usage time levels (41), and late bedtime (42) were associated with an increased risk of NAFLD. Additionally, genetics and age-associated decline in skeletal muscle mass and functional deterioration were also contribute to the NAFLD (43).

We found that 66% of the examined associations had large heterogeneity (>50%) and 48% of them suffered from small-study effects or/and excess significance. Although these features could have resulted from genuine differences across the studies included, the results also suggested that biased results existed in some of the original studies included and subsequently caused concerns in the epidemiological credibility of these meta-analyses. Thus, in the present umbrella review, in accordance with previously published umbrella reviews (24–27), we calculated the levels of the associations between risk/protective factors and NAFLD by examining sample sizes, ES of the largest original study, 95% PIs, heterogeneity, small-study effects, excess significance bias, and the summary ES together.

We found that waist circumference (highly suggestive) and BMI (suggestive) were both risk factors for NAFLD. Obesity, particularly visceral obesity, has long been established as a major risk factor for NAFLD (44). Visceral adipose tissue is known to play an important role in the development of NAFLD by secreting free fatty acids and adipokines (44). In the present study, it was interesting that waist circumference had a higher evidence level than BMI. This could result from the higher heterogeneity between studies of BMI than waist circumference. The lower *P* value of the random-effects model for the association between waist circumference and NAFLD also contributed to the results. Moreover, considering that visceral obesity is a risk factor for a number of complications of metabolic syndrome, assessment of waist circumference may be more sensitive than BMI (45). However, BMI and waist circumference showed conflicting associations with adiposity in different populations (e.g., sex and race) (46). Thus, it is better to assess both BMI and waist circumference as risk factors for the development of NAFLD (45). Indeed, weight loss has been recommended as a key strategy for the improvement of NAFLD (47). In terms of lifestyle change, healthy diet and increased physical activities remain important approaches to the treatment of obesity and its related NAFLD.

In the present study, positive associations were observed for the consumption of sugar-sweetened beverages (highly suggestive) and soft drinks (suggestive) and NAFLD. The consumption of fructose, which is the major sweetener in sugar-sweetened beverages and soft drinks, was strongly suggested as a major dietary risk factor for NAFLD (48). Firstly, increased energy intake and subsequent obesity due to sugar-sweetened beverages and soft drinks could be reasons for the development of NAFLD (49). Secondly, the associations between fructose intake and NAFLD may also be independent of energy intake (50) and/or weight gain (51). Previous studies suggested that fructose intake appears to play an important role in the development of NAFLD by stimulating *de novo* lipogenesis, blocking β-fatty acid oxidation, and improving insulin resistance (48, 52, 53). In recent years, lean NAFLD, a form of NAFLD that occurs among patients considered lean or non-obese, has been the focus of many studies due to its worse outcomes and the more rapid development of cirrhosis than obese NAFLD (54, 55). Although no guidelines are currently available for the treatment of lean NAFLD, a strategy based on weight loss [suggested by the American Association for the Study of Liver Diseases (47)] seems infeasible. However, dietary interventions (such as decreased fructose intake) could be useful as they also aim at improving *de novo* lipogenesis, blocking β-fatty acid oxidation, and reducing insulin resistance. Consumption of high-fructose corn syrup has increased dramatically since the 1970s, mostly in sugar-sweetened beverages and soft drinks (56). There is an urgent need to establish preventive strategies for reducing NAFLD incidence based on dietary fructose control. We also found that consumption of red meat and nuts were positively and negatively associated with NAFLD, respectively, based on weak evidence. However, considering the low levels of evidence of the associations between these two food groups and NAFLD, further high-quality studies are needed.

Both healthy diet and increased physical activities were considered as major lifestyle interventions for the management of NAFLD according to most current guidelines (57). We found that physical activity was a protective factor for NAFLD based on suggestive evidence. Firstly, in line with dietary factors, higher physical activity was associated with NAFLD, partly due to weight loss (31). A previous study found that moderate-to-vigorous physical activity was associated with less subcutaneous and visceral adipose tissues (58). Secondly, apart from weight loss, physical activity itself also has a beneficial association with NAFLD. A previous study found that physical activity was associated with NAFLD independently of obesity (59). The mechanisms underlying these associations are not fully understood. However, a randomized controlled trial suggested that resistance exercise specifically improves intrahepatic lipid content, insulin sensitivity, and metabolic flexibility in NAFLD independently of weight loss (60). Future studies are needed to explore the biologic mechanisms underlying the independent effect of physical activity on NAFLD.

Moreover, we found that former smoking was a lifestyle risk factor for NAFLD based on suggestive evidence. A possible explanation for this association could be that smoking cessation could induce weight gain (61). A previous meta-analysis suggested that smoking cessation is associated with a mean increase in body weight of 4–5 kg after 12 months of abstinence (62). The underlying mechanisms are complicated and not well understood. Firstly, the absence of nicotine increases the rewarding value of food, particularly increasing the intake of food high in sugar and fat (63). Secondly, the change of intestinal microbiota induced by smoking cessation could also partially explain the post-cessation weight gain (64, 65). However, we did not observe a solid association between current smoking and NAFLD. One possible reason could be that NAFLD is a chronic disease and some important information was missing in the original studies assessed in the meta-analysis included (e.g., smoking duration) (14). A short period of smoking may not be sufficient to affect the occurrence of NAFLD. For example, most recently, a large cohort study (not included in our review) found that current smoking status, number of pack-years, and urinary cotinine levels were significantly associated with an increased risk of NAFLD (66). Although the associations between former smoking and NAFLD seem to be robust, considering that the all-cause mortality reduced after quitting smoking (67), smoking cessation is still encouraged.

Finally, among blood biomarkers, we identified that serum fetuin-A level was a risk factor for NAFLD based on highly suggestive evidence while vitamin D level was a protective factor for NAFLD based on suggestive evidence. Serum fetuin-A, which is secreted by both the liver and adipose tissue, can contribute to the development of NAFLD by increasing the secretion of inflammatory cytokines via macrophages (68, 69). Moreover, serum fetuin-A is involved in the development of NAFLD by stimulating key enzymes of hepatic lipid metabolism, such as sterol regulatory element-binding protein-1c (70). However, the association between serum fetuin-B and NAFLD was weak. This could be due to the small number of original studies included. On the other hand, vitamin D deficiency was shown to be a risk factor for NAFLD. Vitamin D has been proven to be beneficial for various steps in the progression and worsening of severity of NAFLD, including improvements in insulin secretion and insulin resistance, adipose tissue inflammation, hepatic inflammation, and hepatic fibrosis (71).

Our study had several limitations. Firstly, we only considered associations between risk/protective factors and NAFLD that have been assessed in meta-analyses of observational studies. Studies that have not been evaluated through meta-analyses were missed. Secondly, considering that many cross-sectional studies were included in the meta-analyses, it was impossible to infer causality. However, few meta-analyses that explored the associations between nutritional, lifestyle, and metabolic factors and NAFLD included cohort studies and/or randomized controlled trials only. More cohort studies and randomized controlled trials are needed to provide more robust pieces of evidence of the associations between risk/protective factors and NAFLD. Thirdly, AMSTAR 2 was used to evaluate the methodologic quality of the included meta-analyses. However, the qualities of the meta-analyses included were low (low: three; critically low: seven). Most of the meta-analyses included were not included in the pre-registered protocol. All the meta-analyses included did not explain their selection of the original study designs for inclusion, provide a list of original studies excluded and justify the exclusions, or report on the sources of funding for the original studies included in their meta-analyses. However, the qualities of the included meta-analyses according to AMSTAR 2 would not affect the evidence grading for the associations between nutritional, lifestyle, and metabolic factors and NAFLD in the present umbrella review. Thus, future high-quality meta-analyses that adhere to the principles of AMSTAR 2 are needed. In addition, we found that all the included meta-analyses were low-quality. It may be due to the fact that the 16 items in the AMSTAR2 involved meticulous information, and some may not be presented in the article. Therefore, we try to contact the corresponding authors to request the details information of methods. However, we did not receive response from the authors. Fourthly, the high vs. low analysis is relatively indefinite and will lose much information in the initial study. For this reason, the guiding significance for the population to improve NAFLD is relatively limited. This bias can be avoided by dose-response analysis, but only eight dose-response meta-analysis were included in this study. Therefore, this result should be interpreted with caution. In conclusion, future dose-response initial studies are needed to interpret the summary estimates of meta-analysis and umbrella review more reasonably. Furthermore, we considered as eligible studies only published studies and did not include unpublished data. Thus, the results could be affected due to data loss. Finally, umbrella review is a method that synthesizes a large number of existing meta-analyses, rather than performing the included meta-analyses from the beginning (72). Thus, we can only conclude on characteristics of participants which have been descripted in the included meta-analyses. However, the information that has not been descripted in the included meta-analyses cannot be summarized (e.g., clinical characteristics of participants and diagnose criteria of NAFLD).

## Conclusions

In summary, our study assessed the evidence on 22 associations between nutritional, lifestyle, and metabolic factors and NAFLD. Without considering genetic influences, generally, unhealthy lifestyle (e.g., unhealthy diet, insufficient physical activity, and former smoking) occurred first and induced subsequent metabolic change (such as obesity). In this process, several anthropometric characteristics and blood biomarkers were identified, such as waist circumference, BMI, serum fetuin-A level, and vitamin D level. The results suggested that lifestyle management should be considered as a major primary preventive strategy for NAFLD. Moreover, considering the low quality of meta-analyses included (assessed using AMSTAR 2) and the limited area of research topics, future high-quality original studies and meta-analyses should be conducted to evaluate the associations between nutritional, lifestyle, and metabolic factors and NAFLD.

## Data Availability Statement

The raw data supporting the conclusions of this article will be made available by the authors, without undue reservation.

## Author Contributions

YZ, KN, and YX contributed to the study conception and design. YX, QW, HD, JL, YL, HS, YJ, QC, and KN contributed to check the data and results. YX and QW contributed to the drafting and revising of the manuscript. KN and YZ contributed to the approval of the final version of the manuscript. All authors contributed to the article and approved the submitted version.

## Funding

This study was supported by grants from the National Key R&D Program of China (No. 2017YFC0907401), the National Natural Science Foundation of China (No. 81903302 and No. 91746205), the LiaoNing Revitalization Talents Program (No. XLYC1907102 and No. XLYC1802095), China Postdoctoral Science Foundation (No. 2018M641752 and No. 2018M641753), Shenyang high level innovative talents support program (No. RC190484), and 345 Talent Project of Shengjing Hospital of China Medical University (No. M0294 and No. M0268).

## Conflict of Interest

The authors declare that the research was conducted in the absence of any commercial or financial relationships that could be construed as a potential conflict of interest.

## Publisher's Note

All claims expressed in this article are solely those of the authors and do not necessarily represent those of their affiliated organizations, or those of the publisher, the editors and the reviewers. Any product that may be evaluated in this article, or claim that may be made by its manufacturer, is not guaranteed or endorsed by the publisher.

## Abbreviations

BMI: Body mass index
ES: Effect size
NAFLD: Non-alcoholic fatty liver disease
PI: Prediction intervals.
