# Supplementary material for: Associations of Nutritional, Lifestyle, and Metabolic Factors With Non-alcoholic Fatty Liver Disease: An Umbrella Review With More Than 380,000 Participants
Source: Front Nutr. 2021 Sep 17;8:642509. doi: 10.3389/fnut.2021.642509 (PMC8484322; doi:10.3389/fnut.2021.642509)
Supplement: Supplementary file 1 [file Table_1.DOCX]

**Supplementary Table 1. Search strategy used in the umbrella review**

| **Literature search strategy in PubMed** |
| --- |
| 1: non-alcoholic fatty liver disease OR NAFLD OR non-alcoholic steatohepatitis OR NASH OR nonalcoholic steatohepatitis OR nonalcoholic fatty liver disease  2: meta-analysis OR systematic review  3: 1 AND 2 |
| **Literature search strategy in EMBASE** |
| 1: non-alcoholic fatty liver disease OR NAFLD OR non-alcoholic steatohepatitis OR NASH OR nonalcoholic steatohepatitis OR nonalcoholic fatty liver disease  2: ‘meta-analysis’ OR ‘systematic review’  3: 1 AND 2 |
| **Literature search strategy in Web of Science** |
| 1: Search TS= (non-alcoholic fatty liver disease OR NAFLD OR non-alcoholic steatohepatitis OR NASH OR nonalcoholic steatohepatitis OR nonalcoholic fatty liver disease)  2: Search TS= (meta-analysis OR systematic review)  3: 1 AND 2 |

**Supplementary Table 2.** Description of the 10 meta-analyses of risk factors for nonalcoholic fatty liver disease.

| **Author (ref)** | **Year** | **Journal** | **Age of participants** | **Sex of participants** | **Study design included in meta-analysis** | **Risk factor** | **Type of**  **effect metric** | **PMID** |
| --- | --- | --- | --- | --- | --- | --- | --- | --- |
| Pang et al | 2015 | World journal of gastroenterology | Adults | Male and female | Cohort, Case-control, Nested case-control and Cross-sectional | Waist circumference, waist-to-hip ratio and body mass index | Odds ratio | 25663786 |
| Du et al | 2017 | Lipids in Health and Disease | Adults and children | Male and female | Case-control and Cross-sectional | The level of serum ferritin | Standardized mean difference | 29197393 |
| Qiu et al | 2017 | Therapeutic Advances in Gastroenterology | Adults | Male and female | Cohort | Physical activity | Relative risk | 28932271 |
| Rezayat et al | 2017 | SAGE Open Medicine | Adults | Male and female | Cohort, Case-control and Cross-sectional | Smoking, current smoking and former smoking | Odds ratio | 29399359 |
| Chen et al | 2018 | Clinical Nutrition | Adults | Male and female | Case-control, Cross-sectional and Prospective | Coffee intake | Relative risk | 30573353 |
| Zhu et al | 2019 | BioMed Research International | Children | Male and female | Case-control and Cross-sectional | Vitamin D levels | Standardized mean difference | 31380438 |
| Chen et al | 2019 | International Journal of Environmental Research and Public Health | Adults and children | Male and female | Cohort, Case-control and Cross-sectional | Sugar-sweetened beverages | Relative risk | 31234281 |
| Liu et al | 2020 | European Journal of Gastroenterology & Hepatology | Adults | Male and female | Prospective cohort, Case-control and Cross-sectional | 25-hydroxyvitamin D and vitamin D level | Standardized mean difference | 31895886 |
| Pan et al | 2020 | International Journal of Environmental Research and Public Health | Adults and children | Male and female | Case-control | Fetuin-A and fetuin-B concentrations | Standardized mean difference | 32326594 |
| He et al | 2020 | The British Journal of Nutrition | Adults | Male and female | Case-control and Cross-sectional | Red meat, soft drinks, nut, whole grains, refined grains, fish, Fruit, vegetables, eggs, dairy and legumes | Odds ratio | 32138796 |

FFQ, food frequency questionnaire; BDHQ, brief-type self-administrated diet questionnaire, N/A, not applicable.

**Supplementary Table 3.** The list of the excluded articles during the process of full text review.

| **No number of cases or sample size for each original study (n = 12)** | |
| --- | --- |
| 1 | Modest alcohol consumption decreases the risk of nonalcoholic fatty liver disease: A meta-analysis of 43175 individuals |
| 2 | Vitamin D and nonalcoholic fatty liver disease |
| 3 | Association between Helicobacter pylori infection and nonalcoholic fatty liver disease: A systematic review and meta-analysis of observational studies |
| 4 | Helicobacter pylori and Risk of Nonalcoholic Fatty Liver Disease |
| 5 | Relationship between Hypothyroidism and Non-Alcoholic Fatty Liver Disease: A Systematic Review and Meta-analysis |
| 6 | The global epidemiology of lean nonalcoholic fatty liver disease: a systematic review and meta-analysis |
| 7 | Prevalence, incidence and risk factors of tamoxifen-related non-alcoholic fatty liver disease: A systematic review and meta-analysis |
| 8 | Marijuana is not associated with progression of hepatic fibrosis in liver disease: a systematic review and meta-analysis |
| 9 | The relationship between obesity and the severity of non-alcoholic fatty liver disease: systematic review and meta-analysis |
| 10 | Fatty acid and non-alcoholic fatty liver disease: Meta-analyses of case-control and randomized controlled trials |
| 11 | Nutritional supplementation for non-alcohol-related fatty liver disease A network meta-analysis |
| 12 | Do Fructose-Containing Sugars Lead to Adverse Health Consequences: Results of Recent Systematic Reviews and Meta-analyses |
| **Intervention study (n = 7)** | |
| 1 | Evidence-Based Exercise Recommendations to Reduce Hepatic Fat Content in Youth: a Systematic Review and Meta-Analysis |
| 2 | Effectiveness of Omega-3 Polyunsaturated Fatty Acids in Non-Alcoholic Fatty Liver Disease: A Meta-Analysis of Randomized Controlled Trials |
| 3 | Effects of probiotics on nonalcoholic fatty liver disease: A meta-analysis |
| 4 | Impact of bacterial probiotics on obesity, diabetes and non-alcoholic fatty liver disease related variables |
| 5 | Metabolic benefits of dietary prebiotics in human subjects: a systematic review of randomised controlled trials |
| 6 | Omega-3 polyunsaturated fatty acid supplementation and non-alcoholic fatty liver disease: A meta-analysis of randomized controlled trials |
| 7 | Association of weight loss interventions with changes in biomarkers of nonalcoholic fatty liver disease: a systematic review and meta-analysis |
| **No data synthesis (n = 10)** | |
| 1 | Review article coffee consumption, the metabolic syndrome and non-alcoholic fatty liver disease |
| 2 | The potential association between periodontitis and non-alcoholic fatty liver disease: a systematic review |
| 3 | Omega-3 fatty acids and nonalcoholic fatty liver disease in adults and children where do we stand |
| 4 | Non-alcoholic fatty liver disease and thyroid dysfunction: a systematic review |
| 5 | Relationship between Diet and Non-alcoholic Fatty Liver Disease: A Review Article |
| 6 | Coffee and non-alcoholic fatty liver disease: Brewing evidence for hepatoprotection? |
| 7 | Celiac Disease, Gluten-Free Diet, and Metabolic and Liver Disorders |
| 8 | Energy and Fructose From Beverages Sweetened With Sugar or High-Fructose Corn Syrup Pose a Health Risk for Some People |
| 9 | Chronic hepatitis B and non-alcoholic fatty liver disease: Conspirators or competitors? |
| 10 | The association of impaired lung function and nonalcoholic fatty liver disease: A systematic review |
| **Duplicated report (n = 2)** | |
| 1 | Adiposity as a risk factor of non alcoholic fat disease: systematic review |
| 2 | Association of serum vitamin D level and nonalcoholic fatty liver disease: a meta-analysis |
| **Meeting abstract** **(n = 14)** | |
| 1 | Association of dietary carbohydrates and sugars with nonalcoholic fatty liver disease: a systematic review |
| 2 | Benefits of low-carbohydrate diet in non-alcoholic fatty liver disease |
| 3 | Impact of Inflammatory Bowel Disease Subtypes and Other Risk Factors on Incidence Rate of Nonalcoholic Fatty Liver Disease: A Systematic Review and Meta-Analysis |
| 4 | Fructose consumption and non-alcoholic fatty liver disease: A systematic review and meta-analysis |
| 5 | Vitamin D and non-alcoholic fatty liver disease: a systematic review and meta-analysis |
| 6 | Moderate alcohol consumption and its effect on fibrosis stage in patients with  non-alcoholic fatty liver disease |
| 7 | Modest alcohol consumption and risk of advanced liver fibrosis in nonalcoholic fatty liver disease: A meta-analysis |
| 8 | Association between diastolic cardiac dysfunction and nonalcoholic fatty liver disease: A systematic review and meta-analysis |
| 9 | Association between testosterone, sex hormone-binding globulin, and nonalcoholic fatty liver disease A systematic review and meta-analysis |
| 10 | Association of non-alcoholic fatty liver disease with thyroid function A systematic review and meta-analysis |
| 11 | Hyperuricemia and risk of nonalcoholic fatty liver disease: A meta-analysis |
| 12 | Type 2 diabetes mellitus and non-alcoholic fatty liver disease: A systematic review and meta-analysis |
| 13 | Relationship between relative skeletal muscle mass and nonalcoholic fatty liver disease: A systematic review and meta-analysis |
| 14 | The association between hypothyroidism and non-alcoholic fatty liver disease: A systematic review and meta-analysis |
